# Supplementary material for: Induced Heteroresistance in Carbapenem-Resistant Acinetobacter baumannii (CRAB) via Exposure to Human Pleural Fluid (HPF) and Its Impact on Cefiderocol Susceptibility
Source: Int J Mol Sci. 2023 Jul 21;24(14):11752. doi: 10.3390/ijms241411752 (PMC10380697; doi:10.3390/ijms241411752)
Supplement: Supplementary file 1 [file ijms-24-11752-s001.zip › Figure S3 EDTA and Boronic .pdf]

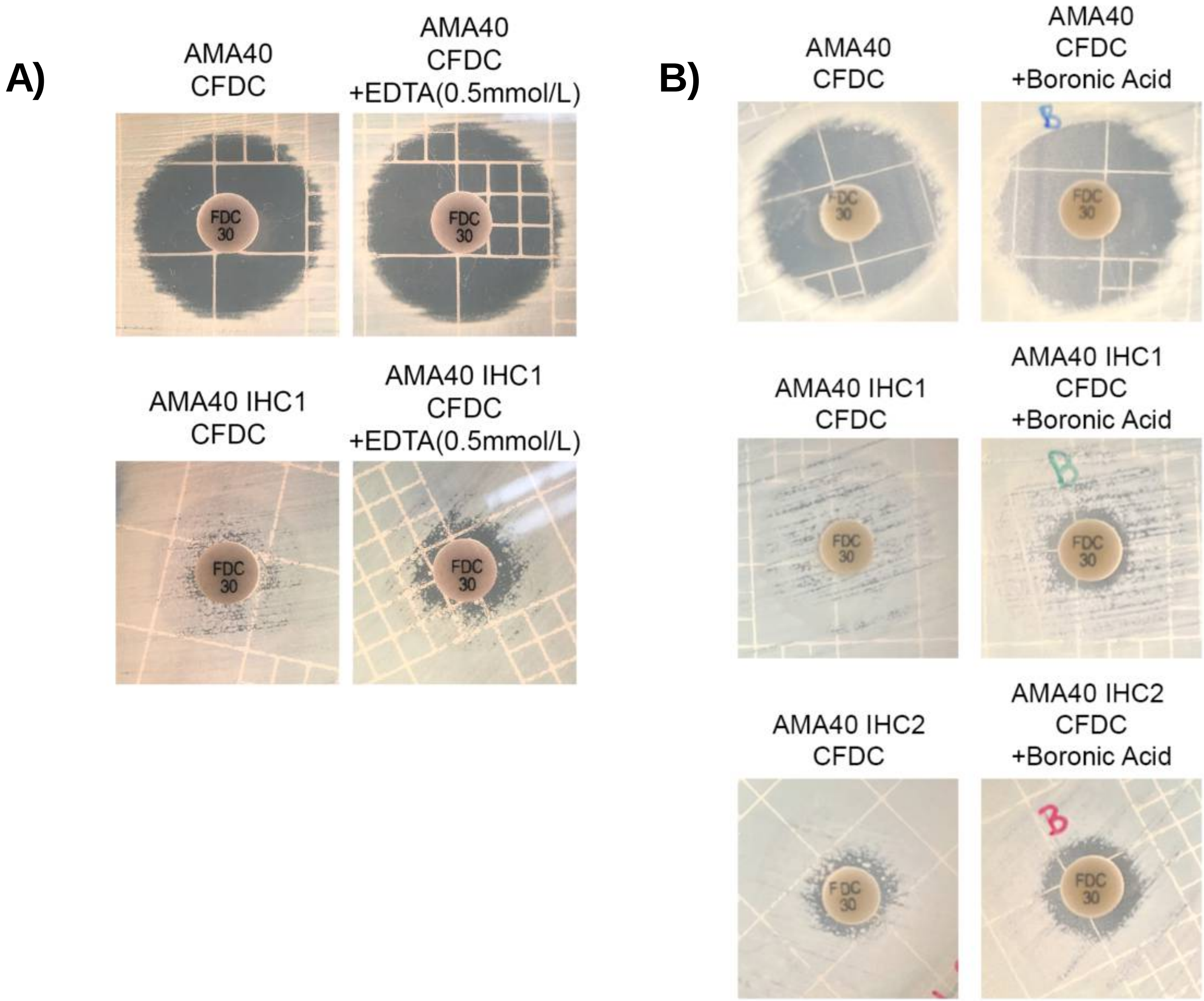

A)
AMA40
CFDC
AMA40
CFDC
+EDTA(0.5mmol/L)
FDC 30
AMA40 IHC1
CFDC
AMA40 IHC1
CFDC
+EDTA(0.5mmol/L)
B)
AMA40
CFDC
AMA40
CFDC
+Boronic Acid
AMA40 IHC1
CFDC
AMA40 IHC1
CFDC
+Boronic Acid
AMA40 IHC2
CFDC
AMA40 IHC2
CFDC
+Boronic Acid
