# Supplementary material for: Induced Heteroresistance in Carbapenem-Resistant Acinetobacter baumannii (CRAB) via Exposure to Human Pleural Fluid (HPF) and Its Impact on Cefiderocol Susceptibility
Source: Int J Mol Sci. 2023 Jul 21;24(14):11752. doi: 10.3390/ijms241411752 (PMC10380697; doi:10.3390/ijms241411752)
Supplement: Supplementary file 1 [file ijms-24-11752-s001.zip › Figure S4.pdf]

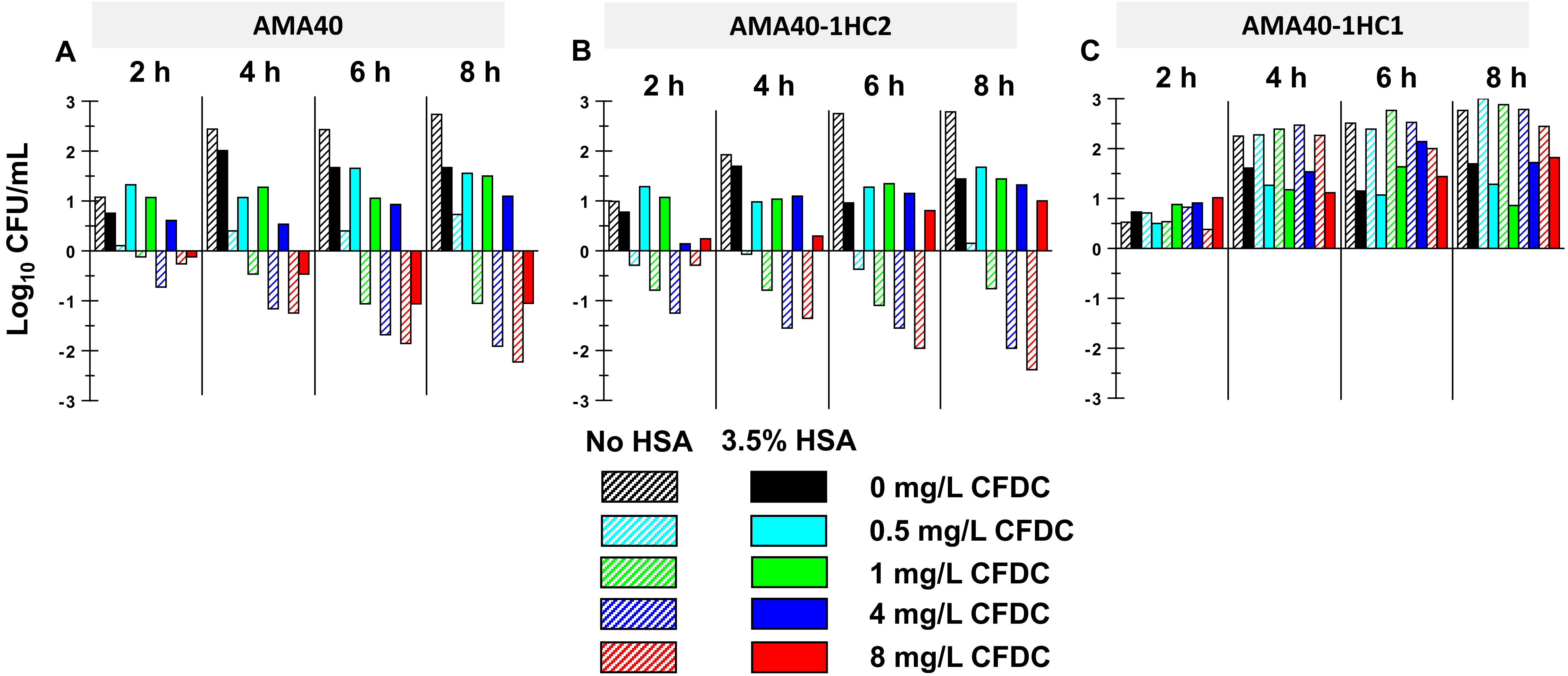

Figure S4. Reduction or increase of bacterial counts (Log_{10} CFU/mL) of (A) *A. baumannii* AMA40, (B) AMA40 IHC2 and (C) AMA40 IHC1 cultured in CAMHB (dashed bars) or CAMHB + 3.5% HSA (solid bars) for the different cefiderocol concentrations evaluated.
