# Supplementary material for: Induced Heteroresistance in Carbapenem-Resistant Acinetobacter baumannii (CRAB) via Exposure to Human Pleural Fluid (HPF) and Its Impact on Cefiderocol Susceptibility
Source: Int J Mol Sci. 2023 Jul 21;24(14):11752. doi: 10.3390/ijms241411752 (PMC10380697; doi:10.3390/ijms241411752)
Supplement: Supplementary file 1 [file ijms-24-11752-s001.zip › Supplementary Material Legends Revised version_MRT.pdf]

Supplementary Material Legends

Figure S1. Effect of HPF on the antimicrobial susceptibility of *A. baumannii* strains AMA40, AMA40 IHC1 and IHC2 to performed cefiderocol (CFDC) susceptibility. Minimum inhibitory concentration (MIC) was performed following manufacturer's recommendations (Liofilchem S.r.l., Italy).

Figure S2. Effect of β-lactamase inhibitors (avibactam, relebactam, or zidebactam) on the antimicrobial susceptibility of *A. baumannii* strains AMA40, AMA40 IHC1 and IHC2 to performed cefiderocol (CFDC) susceptibility. Minimum inhibitory concentration (MIC) on cation adjusted Mueller Hinton agar supplemented with 4 µg/mL avibactam (AVI), relebactam (REL), or zidebactam (ZID) of was performed following manufacturer's recommendations (Liofilchem S.r.l., Italy).

Figure S3. Effect of EDTA **(A)** and boronic acid **(B)** on the antimicrobial susceptibility of *A. baumannii* strains AMA40, AMA40 IHC1 and IHC2 to performed cefiderocol (CFDC) susceptibility. Minimum inhibitory concentration (MIC) was performed following manufacturer's recommendations (Liofilchem S.r.l., Italy).

Figure S4. Reduction or increase of bacterial counts (Log_{10} CFU/mL) of (A) *A. baumannii* AMA40, (B) AMA40 IHC2 and (C) AMA40 IHC1 cultured in CAMHB (dashed bars) or CAMHB + 3.5% HSA (solid bars) for the different cefiderocol concentrations evaluated.

Table S1. Mutations identified in the AMA40 IHC1 and IHC2 strains compared to the parental strain.

Table S2. Minimal Inhibitory Concentrations of cefiderocol (CFDC) performed using cefiderocol MTS strips (Liofilchem S.r.l., Italy) on cation-adjusted Mueller Hinton Agar (CAMHA) alone or

supplemented with the different β-lactamase inhibitors: 4 µg/mL of avibactam (AVI), relebactam (REL), or zidebactam (ZID).

Table S3. Minimal Inhibitory Concentrations of cefiderocol (CFDC) performed using cefiderocol MTS strips (Liofilchem S.r.l., Italy) on cation-adjusted Mueller Hinton Agar (CAMHA) alone or supplemented with 300 mg/L $ZnSO_4$.

Table S4. qRT- PCR primers used in this study.
