# Supplementary material for: Induced Heteroresistance in Carbapenem-Resistant Acinetobacter baumannii (CRAB) via Exposure to Human Pleural Fluid (HPF) and Its Impact on Cefiderocol Susceptibility
Source: Int J Mol Sci. 2023 Jul 21;24(14):11752. doi: 10.3390/ijms241411752 (PMC10380697; doi:10.3390/ijms241411752)
Supplement: Supplementary file 1 [file ijms-24-11752-s001.zip › Table S2.pdf]

Table S2. Minimal Inhibitory Concentrations of cefiderocol (CFDC) performed using cefiderocol MTS strips (Liofilchem S.r.l., Italy) on cation-adjusted Mueller Hinton Agar (CAMHA) alone or supplemented with the different DBOs.

Strain	Condition			
	CAMHA	CAMHA+AVI (4ug/ml)	CAMHA+REL (4ug/ml)	CAMHA+ZID (4ug/ml)
AMA40	0.38	0.50	0.38	0.75
AMA40 IHC1	>256	1.00	0.50	0.75
AMA40 IHC2	8	1.00	0.50	0.75

AVI: avibactam, REL: relebactam, ZID: zidebactam
