# Supplementary material for: Induced Heteroresistance in Carbapenem-Resistant Acinetobacter baumannii (CRAB) via Exposure to Human Pleural Fluid (HPF) and Its Impact on Cefiderocol Susceptibility
Source: Int J Mol Sci. 2023 Jul 21;24(14):11752. doi: 10.3390/ijms241411752 (PMC10380697; doi:10.3390/ijms241411752)
Supplement: Supplementary file 1 [file ijms-24-11752-s001.zip › Table S4.pdf]

qRT-PCR Primer Table for AMA40, IHC1, and IHC2		
Primer Name	Relation	5’-3’ Sequence
bla$_{ADC}$ F *(ampC)*	β-lactamase	TGCCAACCTTAACCCACA
bla$_{ADC}$ R *(ampC)*	β-lactamase	AGTTTGTAACGTTGCCGG
bla$_{NDM-1}$ F	β-lactamase	GAAGCTGAGCACCGCATTAG
bla$_{NDM-1}$ R	β-lactamase	AACCAGATCGCCAAACCGTT
bla$_{ompA}$ F	β-lactamase	TCTTGGTGGTCACTTGAAGC
bla$_{ompA}$ R	β-lactamase	ACTCTTGGCTTGTGGAGCA
bla$_{carO}$ F	Resistance	GGCGGATGAAGCTGTTGTTC
bla$_{carO}$ R	Resistance	GCCATAACAAAGCACCACCG
bauA F	Iron uptake	AAATGTTTGGCCGCGTTGAGGT
bauA R	Iron uptake	CAATCGTGCAAACGGTTCATCAGC
bfmR F	Biofilm	CGATGGTAACCGTGCAATTCGT
bfmR R	Biofilm	ATCGTCTGCACCCATTTCCAGA
bfnH F	Iron uptake	ACTGCGACTCGTACACCAAA
bfnH R	Iron uptake	ACTTTACGACCTGCCGTAGC
csuAB F	Biofilm	CAGGCTGTACTGTAGGTG
csuAB R	Biofilm	CAGGATCTGTTCCGTCAC
csuB F	Biofilm	GCAAAGGTTCTTGGAATATCCGT
csuB R	Biofilm	TGGACGAACCATTCTTCATACGT
csuE F	Biofilm	TGGACAAAGTGTATCGCCGG
csuE R	Biofilm	ACACCCCGATTCCCACAATC
exbD F	Iron uptake	GCAGGTCAAGGTGCGGTTA
exbD R	Iron uptake	CGCGAGACATTACCTGAGCA
pirA F	Iron uptake	GTCTATGGCTTTTGCTGCACA
pirA R	Iron uptake	GCGATTGCTTCACTTGCTCT
pilT F	Biofilm	CTTTGGTCTAGTGTGGTCATGC
pilT R	Biofilm	AAACAAAGTCGCGCAAATG
piuA F	Iron uptake	ATGGCGCAAGAAGCAGTTTC
piuA R	Iron uptake	TGTCTTTGAGAGGAGCCACG
tonB3 F	Iron uptake	AAGCCAAAGCCAGTGGTTC
tonB3 R	Iron uptake	CTCCACTGTACACCCGAACC
