# Supplementary figures and images for: Induced Heteroresistance in Carbapenem-Resistant Acinetobacter baumannii (CRAB) via Exposure to Human Pleural Fluid (HPF) and Its Impact on Cefiderocol Susceptibility
Source: Int J Mol Sci. 2023 Jul 21;24(14):11752. doi: 10.3390/ijms241411752 (PMC10380697; doi:10.3390/ijms241411752)

AMA40

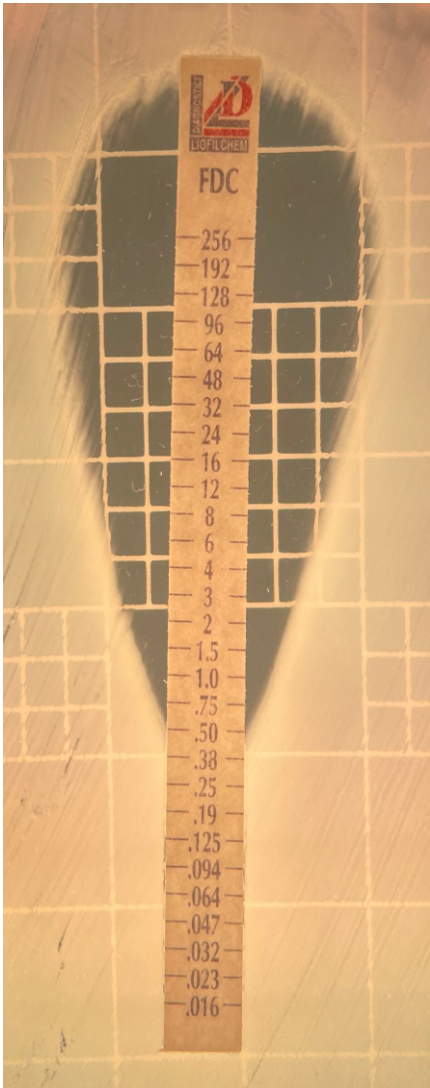

AMA40 IHC1

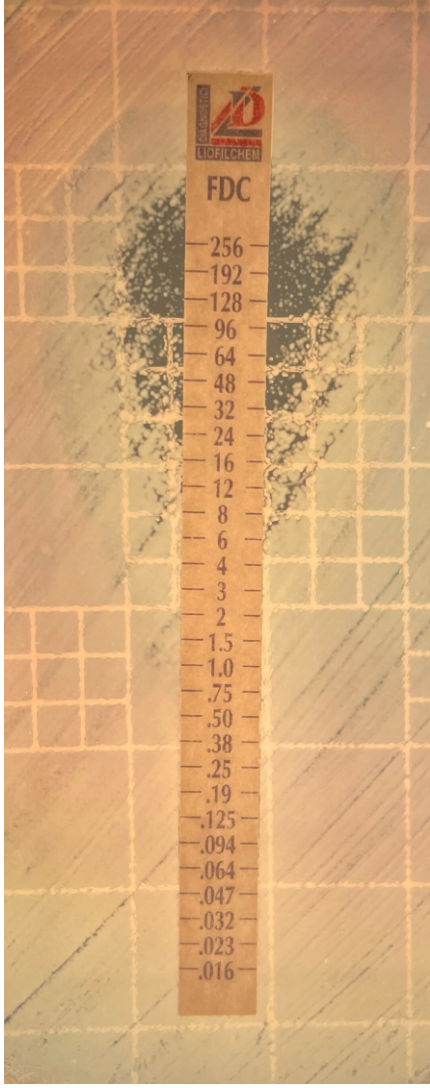

AMA40 IHC2

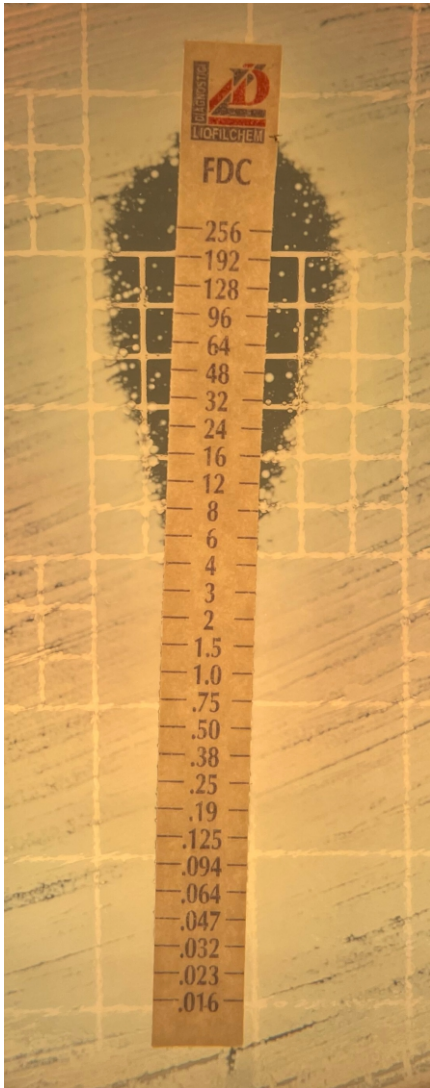

Supplement: Supplementary file 1 [file ijms-24-11752-s001.zip › Figure S1.pdf]
